# When environmentally persistent pathogens transform good habitat into ecological traps

**DOI:** 10.1098/rsos.160051

**Published:** 2016-03-23

**Authors:** Clinton B. Leach, Colleen T. Webb, Paul C. Cross

**Affiliations:** 1 Department of Biology, Graduate Degree Program in Ecology, Colorado State University, Fort Collins, CO, USA; 2 US Geological Survey, Northern Rocky Mountain Science Center, Bozeman, MT, USA

**Keywords:** disease, metapopulation, habitat quality, environmental transmission, ecological trap

## Abstract

Habitat quality plays an important role in the dynamics and stability of wildlife metapopulations. However, the benefits of high-quality habitat may be modulated by the presence of an environmentally persistent pathogen. In some cases, the presence of environmental pathogen reservoirs on high-quality habitat may lead to the creation of ecological traps, wherein host individuals preferentially colonize high-quality habitat, but are then exposed to increased infection risk and disease-induced mortality. We explored this possibility through the development of a stochastic patch occupancy model, where we varied the pathogen’s virulence, transmission rate and environmental persistence as well as the distribution of habitat quality in the host metapopulation. This model suggests that for pathogens with intermediate levels of spread, high-quality habitat can serve as an ecological trap, and can be detrimental to host persistence relative to low-quality habitat. This inversion of the relative roles of high- and low-quality habitat highlights the importance of considering the interaction between spatial structure and pathogen transmission when managing wildlife populations exposed to an environmentally persistent pathogen.

## 1. Introduction

Many prominent and problematic wildlife diseases are caused by pathogens that can persist, and remain infectious, for long periods of time in the environment. Examples include chronic wasting disease [1], anthrax (*Bacillus anthracis* [2]), plague (*Yersinia pestis* [3]) and white nose syndrome (*Pseudogymnoascus destructans* [4]), among others. This environmental persistence creates environmental pathogen reservoirs from which susceptible hosts can become infected without direct contact with an infectious individual. This additional transmission pathway can have important consequences for disease dynamics, with models showing that increased environmental longevity generally facilitates increased pathogen persistence and spread relative to direct transmission alone [5]; [6,7].

Since environmental transmission is necessarily a spatial process, its role in disease dynamics may further depend on the spatial structure of the host population. For many wildlife hosts with environmentally persistent pathogens (e.g. prairie dog colonies affected by plague [8]), this spatial structure is well described as a metapopulation, where host populations occupy small patches of suitable habitat in a highly fragmented landscape. Taking a metapopulation perspective then, we expect that host population structure and movement will be influenced by heterogeneity in the quality of these habitat patches. Indeed, the quality of a habitat patch can affect its extinction and colonization rates, as well as its contribution to the colonization of other empty patches [9]. These processes will influence how a pathogen spreads, where environmental pathogen reservoirs get established, and the resulting effects on host occupancy and population size.

We expect that high-quality habitat patches, which are more attractive and support greater host density than lower quality habitat, might be more likely to form pathogen reservoirs and support consistently infectious populations. As these reservoirs are undetectable to the host, we predict that high-quality patches will continue to attract—and infect—susceptible immigrants. The resulting fitness consequences of increased disease risk would then effectively create an ecological trap for migrant hosts preferentially selecting high-quality habitat [5,10]. In addition, the greater stability of high-quality patches may further facilitate pathogen spread by positioning high-quality patches as metapopulation-scale superspreaders [11]. Similarly, we expect that low-quality patches will be less likely to develop pathogen reservoirs and may serve as refuges where susceptible hosts can escape infection.

The combination of these effects may then lead to the creation of a sort of management trap at the metapopulation-scale, where the conservation or restoration of otherwise high-quality habitat in the metapopulation actually reduces total host population size relative to actions focused on low-quality habitat. Thus, understanding how pathogen transmission interacts with patterns of habitat quality is critical to managing these systems. In this study, we explore this interaction and its influence on host occupancy and overall population size. We specifically focus on the relative importance of high- and low-quality habitats to host persistence in the presence of pathogens with different levels of environmental longevity.

## 2. Material and methods

### 2.1 Stochastic patch occupancy model

To address the above questions, we developed a model based on the stochastic patch occupancy model (SPOM) framework. This framework, based on a well-developed body of theory [12,13], models the occupancy state of discrete patches of suitable habitat. The SPOM approach is flexible and able to capture realistic features of a landscape, including spatial structure and heterogeneity in habitat quality, while remaining relatively tractable [13]. To incorporate disease processes, we expand on existing SPOM models [12,13] and allow each patch to be in one of three possible states: occupied by susceptible hosts (*S*), occupied by infectious hosts (*I*) and unoccupied by the host (∅). Transitions between states are governed by host colonization and extinction rates, and a susceptible population can become infected either through direct contact with infectious immigrants, or through a local environmental pathogen reservoir (table 1).

**Table 1.** State transitions and their rates for patch *i* in a metapopulation simulation. (*S* denotes occupied by susceptible hosts, *I* denotes occupied by infectious hosts and ∅ denotes unoccupied by the host. All patches are characterized by an environmental transmission rate, *γ*(*τ*_*i*_), where *τ*_*i*_ is the time since the patch was last occupied by infectious individuals.)

state transition	process	rate
S→I	infection (from contact or reservoir)	*δC*_*Ii*_+*γ*(*τ*_*i*_)
S→∅	extinction (of susceptible)	*E*_*Si*_
I→∅	extinction (of infectious)	*E*_*Ii*_
∅→S	colonization (by susceptibles)	*C*_*Si*_
∅→I	colonization (by infectious)	*C*_*Ii*_

We assume that the host population size supported by a patch is proportional to its quality (*A*_*i*_), and that a proportion, *ν*, of hosts survive when a population is infected (i.e. the size of infectious populations is reduced by a factor of *ν*). Building on the framework developed by [12] then, the rates at which patch *i*, with quality (or equivalently population size) *A*_*i*_, is colonized by susceptible and infectious individuals, respectively, is given by its connectivity:

2.1 $$C_{Si}(t)=A_{i}^{\xi_{\mathrm{im}}}\sum_{i\neq j}\phi_{j}(S,t)A_{j}^{\xi_{\mathrm{em}}}\exp(-Dd_{ij}),$$

and

2.2 $$C_{Ii}(t)=A_{i}^{\xi_{\mathrm{im}}}\sum_{i\neq j}\phi_{j}(I,t){\left(\nu A_{j}\right)}^{\xi_{\mathrm{em}}}\exp(-Dd_{ij}),$$

where *ϕ*_*j*_(*X*,*t*) is an indicator function that is 1 if patch *j* is in state *X* at time *t* and is 0 otherwise; *ξ*_im_ and *ξ*_em_ control how rates of immigration and emigration, respectively, scale with patch quality/population size; *D* is the inverse of the host’s mean dispersal distance and *d*_*ij*_ is the effective distance between patches *i* and *j*. Essentially, *C*_*Xi*_ sums the colonization effort (or propagule pressure) from all patches in state *X* to focal patch *i*.

The state-dependent extinction rates of populations on patch *i* are then given by

2.3 $$E_{Si}=\frac{\mu }{A_{i}^{\alpha }}$$

and

2.4 $$E_{Ii}=\frac{\mu }{{\left(\nu A_{i}\right)}^{\alpha }},$$

where *μ* is the extinction rate of a patch of unit quality, and *α* controls how extinction rate scales with population size. When an infectious population goes extinct, we assume that the hosts leave behind an infectious pathogen reservoir on the patch.

Susceptible host populations can become infected through two different routes: via direct contact with infectious immigrants and via an environmental reservoir left behind by a previously infectious population. Infectious immigrants arriving on a susceptible patch (at rate *C*_*Ii*_) infect the resident population with probability *δ*, while susceptible populations occupying a contaminated patch become infected at rate

2.5 $$\gamma (\tau_{i})=\gamma_{0}\exp(-r\tau_{i}),$$

where *γ*_0_ is the initial infection rate from the pathogen reservoir, *τ*_*i*_ is the time since last infectious occupancy ($\tau_{i}=\infty$, and thus *γ*(*τ*_*i*_)=0, if the patch has never been occupied by infectious hosts) and *r* is the pathogen’s decay rate in the environment, determined by the pathogen’s longevity, *β* (defined as its half-life in the environment relative to the expected residence time of a population on a unit quality patch):

2.6 $$r=\frac{\mu \log(2)}{\beta }.$$

### 2.2 Model parametrization

The model was parametrized according to the following assumptions (table 2): (i) the rate of emigration from a patch increases with the local population size, while the extinction rate of a patch decreases with population size (*ξ*_em_=0.5, *α*=1 [12]); (ii) migrants preferentially select high-quality habitat, that is, the rate at which a patch is colonized scales with quality (*ξ*_im_=0.5 [12]); and (iii) patches are arranged in a lattice and only accessible from their four nearest neighbours (*d*_*ij*_=1 for all *i*, *j* neighbours). In addition to these assumptions, we chose parameters so that without infection, approximately 0.75 of the patches were occupied (*μ*=0.1, *D*=2). We varied infectious survival (*ν*), probability of direct transmission (*δ*), and environmental longevity to explore the dynamics of a range of possible pathogens.

**Table 2.** Parameters of the SPOM model, their meaning and the values, or range of values, assigned.

parameters	interpretation	value(s)
*ξ*_im_	effect of patch quality on immigration (preference for high-quality habitat)	0, 0.5
*ξ*_em_	effect of population size on emigration	0.5
*D*	inverse of mean dispersal distance	2 (lattice), 5 (full)
*d*_*ij*_	distance between patch *i* and *j*	1∀*i*,*j* neighbours (lattice)
		1∀*i*≠*j* (full)
*μ*	extinction rate of unit quality patch	0.1
*ν*	infectious survival	0.1–1
*α*	strength of environmental stochasticity	1
*δ*	probability of direct infection	0–0.9
*γ*_0_	initial rate of infection from reservoir patch	0.5
*β*	pathogen longevity (half-life in environment, relative to 1/*μ*)	0.3–3

To examine the effects of a less rigid spatial structure, we also implemented a model in which all patches are equally accessible from all other patches (i.e. *d*_*ij*_=1, for all *i*≠*j*, in which case average migration distance was adjusted to *D*=5 to maintain roughly equivalent occupancy). The lattice and fully-connected models book-end the range of possible landscape structures, with most realistic metapopulations lying somewhere in between.

### 2.3 Simulation studies

To explore the relative influence of low- and high-quality habitat on host and pathogen dynamics, we simulated the spread of a range of pathogens in metapopulations with patch quality distributions that favour either high- or low-quality habitat. These two distributions were chosen to explore a possible conservation scenario in which managers might need to prioritize habitat for preservation or restoration. To capture the consequences of shifting priorities to either low- or high-quality habitat, we generated patch quality from uniform distributions with equal variance and limits shifted to favour either low- or high-quality habitat (yielding distributions with qualities ranging from 0.23 to 1.24 and from 0.76 to 1.77). We then implemented a full-factorial set of simulations on each quality distribution in which infectious survival (*ν*) and probability of direct transmission (*δ*) were each varied over 10 values (ranging from 0.1 to 1, and 0 to 0.9, respectively). In addition, pathogen longevity (*β*) was varied over three values (ranging from 0.32 to 3.2).

For each combination of disease parameters (*ν*, *δ* and *β*), habitat quality distribution (high or low) and landscape structure (lattice or fully connected), we ran 100 replicate simulations in which qualities for 100 patches were drawn from a uniform distribution on the given habitat quality range (above). In each case, an entirely susceptible population was simulated until it reached an approximate steady state, at which point a randomly chosen occupied patch was infected. The state of the metapopulation was then tracked for 5000 time steps. We recorded the proportion of patches in each state at the end of each simulation and also computed the mean total population size of susceptible and infectious hosts over the last 500 time steps of the simulation to get an estimate of the equilibrium state (summing the local population sizes for all occupied patches, assuming a local population size of *A*_*i*_ for occupied susceptible patches and *νA*_*i*_ for occupied infectious patches).

To more carefully explore the role of habitat quality in the occupancy of a given patch, we performed an additional simulation experiment on metapopulations with patch qualities evenly spaced over the full range explored above (from 0.2 to 1.8). In these simulations, we varied longevity over the values used above and explored the consequences of host preference for high-quality habitat (and the corresponding potential for ecological traps) by choosing two different values for *ξ*_im_ (with *ξ*_im_=0.5 indicating preference for high-quality habitat, and *ξ*_im_=0 indicating that migrants select all patches with equal probability). To isolate the effects of habitat preference and pathogen longevity, we fixed the direct infection probability (*δ*=0.5), infectious survival (*ν*=0.2) and landscape structure (lattice). We performed 100 replicate simulations for each set of model parameters (three values of pathogen longevity and two values of *ξ*_im_) and recorded the proportion of time each patch spent occupied by susceptible and infectious hosts, and the final total host population size.

Continuous time stochastic simulations of the above model were implemented in the R language [14] using the Gillespie algorithm [15]. Code is available from the project’s Github repository [16].

## 3. Results

**Figure 1.**
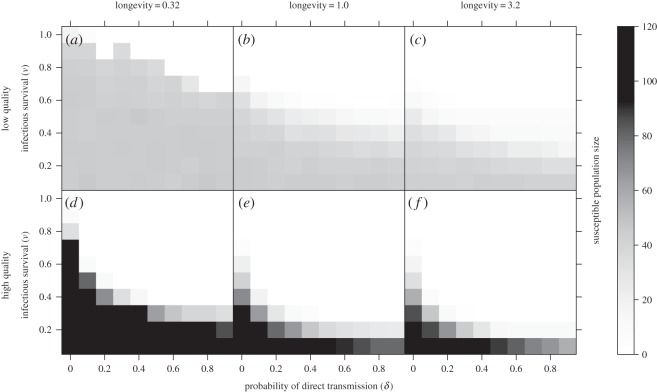
Median total susceptible population size as a function of infectious survival (*ν*), and the probability of direct transmission (*δ*). Panel columns show low- (0.3, *a*,*d*), medium- (1.0, *b*,*e*) and high- (3.2, *c*,*f*) pathogen longevities, while rows show low-quality (ranging from 0.23 to 1.24, *a*–*c*) and high-quality (ranging from 0.76 to 1.77, *d*–*f*) patch quality distributions. Darker shading corresponds to larger population sizes, while white indicates that no susceptible host populations persist (i.e. all host populations become infected).

A wide range of disease dynamics were observed over the range of parameters and habitat quality distributions explored. The ability of the pathogen to spread (and of susceptible hosts to persist) was influenced most strongly by infectious survival, with high infectious survival (i.e. low disease-induced mortality) facilitating widespread infection (in which no susceptible host populations persist), and very low infectious survival (i.e. high disease-induced mortality) facilitating disease-free dynamics or intermediate spread (figure 1). Pathogens with higher environmental longevities were generally able to invade and spread more easily, even with low infectious survival (figure 1*c*,*f*). The distribution of habitat quality in the metapopulation also played an important role in determining disease dynamics. Prioritizing high-quality habitat facilitated widespread infection for a larger range of parameters (figure 1*a*–*c* versus *d*–*f*). Metapopulations with a fully-connected structure exhibited qualitatively similar patterns, but with generally easier pathogen spread (electronic supplementary material, figure S1).

**Figure 2.**
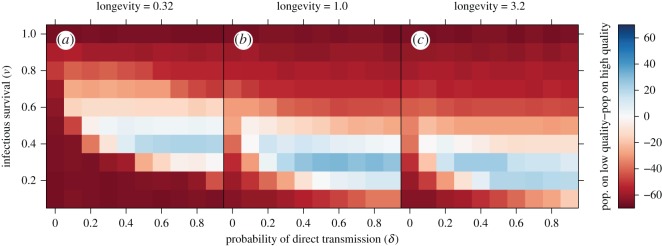
Differences between the median total population size of hosts on low-quality patch distributions (quality between 0.23 and 1.24) and high-quality patch distributions (quality between 0.76 and 1.77) as a function of infectious survival (*ν*) and the probability of direct transmission (*δ*). Reds indicate that high-quality habitat distributions support higher median host population sizes than low-quality habitat, primarily corresponding to pathogens with either very limited or very extensive spread. Conversely, blues indicate higher median host population sizes on low-quality habitat distributions, corresponding to pathogens with intermediate levels of spread. Panel columns show low- (0.3, *a*), medium- (1.0, *b*) and high- (3.2, *c*) pathogen longevities.

**Figure 3.**
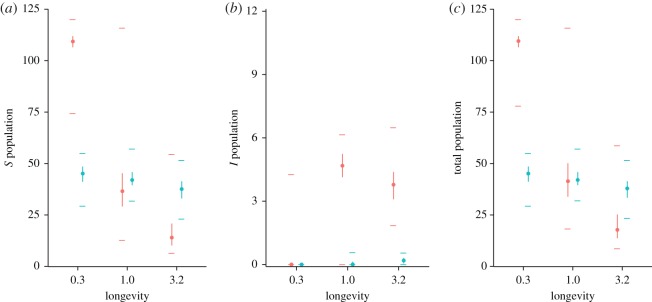
Boxplots showing the range, over 100 replicate simulations, of susceptible (*a*), infectious (*b*) and total (*c*) host population sizes for high-quality (red) and low-quality (blue) habitat distributions. Infectious survival (*ν*) and direct transmission rate (*δ*) are fixed at values of 0.2 and 0.5, respectively. Coloured points show medians, while vertical lines indicate the inner-quartile ranges, with horizontal lines indicating the minimum and maximum. At low longevities, this pathogen is unable to invade and high-quality habitat supports larger populations of susceptible hosts. However, low-quality habitat is better able to maintain these susceptible hosts as the pathogen’s longevity increases, leading to larger total population sizes.

For two large regions of parameter values, high-quality patch distributions had a net positive effect on total host population size relative to low-quality distributions (red shaded regions in figure 2). In the first region (bottom left corner of the panels in figure 2), corresponding to pathogens that were unable to invade owing to low infectious survival and weak transmission, high-quality habitat distributions supported larger total host populations than low-quality distributions owing to the greater stability and connectivity afforded by high-quality habitat (e.g. figure 3 for *β*=0.3). In the second region (top portion of panels in figure 2), corresponding to pathogens that were able to spread widely (i.e. at a pandemic level) owing to high infectious survival and strong transmission (from either environmental or direct transmission), high-quality distributions again supported larger total host populations than low-quality distributions.

However, for pathogens falling between these extremes (e.g. with intermediate infectious survival), having low-quality habitat was a net benefit to the host, with low-quality distributions supporting larger total population sizes than high-quality distributions (blue regions of figure 2). The spread of these pathogens was strongly influenced by the habitat quality distribution, with low-quality habitat limiting disease spread and better maintaining susceptible occupancy relative to high-quality habitat (figure 1). As an example, for a particular pathogen in this region (*ν*=0.2, *δ*=0.5 and *β*=3.2, figure 3), we see that low-quality habitat distributions supported very small infectious populations and moderate susceptible populations. By contrast, high-quality habitat distributions better facilitated the maintenance and spread of infection, substantially reducing the population of susceptibles and thereby the total population size (figure 3). In this case, high-quality habitat served as a sort of metapopulation-level trap, that is, otherwise beneficial habitat that is detrimental to the overall host population in the presence of disease. As pathogen longevity increased, the range of the parameter space where we observed this metapopulation-level trap shifted towards more virulent pathogens. Again, metapopulations with a fully-connected structure displayed similar patterns (electronic supplementary material, figures S2, S3).

**Figure 4.**
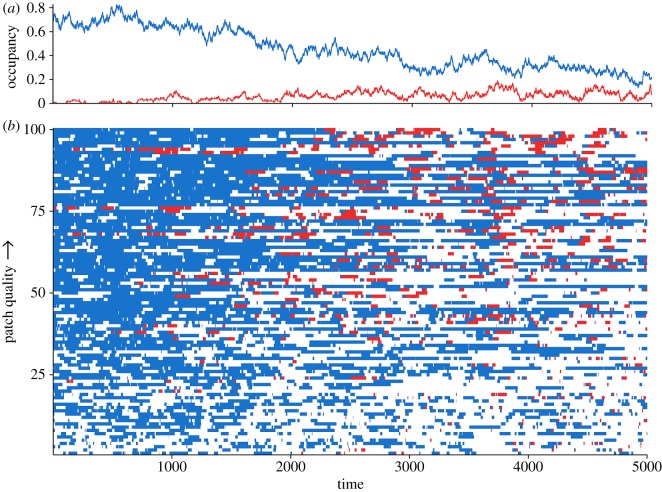
Results from a single representative simulation with habitat quality uniformly distributed between 0.23 and 1.77, and a pathogen with an infectious survival of *ν*=0.2, a direct transmission rate of *δ*=0.5, and an environmental longevity of *β*=3.2. (*a*) shows total proportion of patches occupied by susceptible (blue) and infectious (red) hosts over time; (*b*) shows the state—susceptible (blue), infectious (red) or unoccupied (white)—of individual patches through time. Patches are stacked vertically with the lowest quality at the bottom and the highest quality at the top. Note the overall more consistent infectious occupancy on high-quality habitat and the only very brief spurts of infectious occupancy on low-quality habitat.

To better understand the mechanisms behind these effects, we further examined patch-level occupancy for parameters in this intermediate range (*ν*=0.2 and *δ*=0.5). Because of their lower extinction rate and higher recolonization rate, high-quality patches were more consistently occupied than low-quality patches (figure 4; electronic supplementary material, figures S4, S5). However, because of this stability, disease risk, measured by the mean proportion of time a patch spent occupied by infectious hosts, increased with patch quality (electronic supplementary material, figure S5). Indeed, as an epidemic progressed, high-quality patches increasingly supported infectious occupants, while low-quality patches supported infectious hosts only briefly and infrequently (figure 4). In addition, migrant preference for high-quality habitat (i.e. *ξ*_im_=0.5) increased this disease risk further (electronic supplementary material, figure S5).

These qualitative trends are consistent across both landscape structures, but the effects of quality and preference are more pronounced for the fully-connected case (electronic supplementary material, figures S6, S7). In the fully connected model, high-quality patches attract infectious colonists from the entire metapopulation, not just the local neighbourhood, and thus are more consistently infected than in the lattice model (electronic supplementary material, figure S7).

## 4. Discussion

This work demonstrates that the distribution of habitat quality in a metapopulation can have substantial impacts on the dynamics of a variety of environmentally transmitted pathogens and the resulting size and stability of the host population. In particular, prioritizing the preservation or restoration of high-quality habitat can enhance the spread and impact of environmentally persistent pathogens. In systems with endemic but not widespread disease, these patches with many resources may become metapopulation-scale management traps that are normally beneficial and attractive to hosts, but become a net drain on the metapopulation owing to the impacts of disease. This suggests that the presence of a disease can lead to a trade-off between otherwise high-quality habitat that facilitates metapopulation stability at the expense of increased disease spread, and low-quality habitat that hinders disease spread at the expense of metapopulation stability.

The net consequences of this trade-off for the host metapopulation depend on the characteristics of the disease. Broadly, we can divide the observed dynamics into three different scenarios: limited, intermediate and widespread infection (possibly leading to host extinction). High-quality habitats are a net benefit to the total host population when pathogen spread is either limited or is high enough that both high- and low-quality patches are affected by disease. In these cases, the connectivity and stability of high-quality patches facilitate larger total population sizes with little additional pathogen spread.

There is an intermediate scenario, however, where low-quality habitats are more likely to remain uninfected and as a result boost the total population size, while high-quality patches that are heavily infected tend to reduce the total population size. In this scenario, the ability of pathogens to invade and spread is influenced substantially by the colonization and extinction rates of both high- and low-quality habitat, and the contrasting roles that these habitat types play in the metapopulation.

### 4.1 Role of high-quality habitat

Because high-quality habitat supports larger local host population sizes, these populations are less susceptible to environmental stochasticity and thus experience less frequent extinction events. Owing to this additional stability, high-quality habitat is better able to both maintain a consistent pool of susceptible hosts to which infection can spread, and support infectious host populations under disease-induced mortality. As a result, the probability of infectious occupancy (i.e. proportion of time occupied by infectious hosts) increases with patch quality (electronic supplementary material, figures S5, S7). From the perspective of a migrant susceptible host then, selection of high-quality habitat leads to increased disease risk relative to other habitats, either because the patch is probably already occupied by infectious hosts, or because the local susceptible hosts are likely to soon become infected (either through the presence of an environmental reservoir or direct contact).

Thus, if high-quality habitat is preferred by migrants (i.e. *ξ*_im_>0), it forms an ecological trap at the individual scale, that is, preferentially selected habitat that reduces individual fitness relative to other habitats [10]. Moreover, host preference for high-quality habitat further exacerbates this increase in disease risk (electronic supplementary material, figures S5, S7) by increasing the rate of pathogen import from infectious migrants also selecting high-quality habitat. The magnitude of this effect depends on the landscape structure and the scale at which individuals sense and select habitat. In a fully connected metapopulation, where high-quality patches attract migrants from the entire landscape, the effect of preference on the relationship between quality and disease risk is the most pronounced (electronic supplementary material, figure S7). However, the fact that the effect persists in a lattice metapopulation (electronic supplementary material, figure S5), where migrants can only select among the four neighbouring patches, suggests that host preference for high-quality habitat probably serves to further increase the disease risk on high-quality patches even in more realistic landscapes. This echoes results from the contact network literature which show that highly connected nodes have a higher risk of becoming infected [17,18].

The relatively low extinction rate on high-quality patches, and the resulting increased probability of infectious occupancy, helps to create a relatively stable platform from which the pathogen can spread through the rest of the metapopulation. Indeed, for pathogens with intermediate spread, high-quality patches are effectively metapopulation scale superspreaders [11,19]. This superspreader role helps to maintain infectious occupancy throughout the metapopulation, which, when coupled with preference for high-quality habitat, feeds back on high-quality patches to ensure a steady stream of new infectious colonists. Through these two interacting processes—the ecological trap created by the preference of individuals for high-quality habitat, and the superspreader role emerging from the increased connectivity and stability generated by high-quality habitat—the presence of high-quality patches serves to substantially increase pathogen spread, even for more virulent pathogens (figures 1 and 3). The consequences of widespread disease-induced mortality can then outweigh the positive consequences of increased metapopulation connectivity and stability, leading to the creation of a metapopulation-scale trap and a net decrease in total host population size on high-quality habitat distributions (figures 2 and 3).

### 4.2 Role of low-quality habitat

In contrast with high-quality habitat, low-quality patches help to limit pathogen spread and increase susceptible occupancy in the metapopulation (figures 1 and 3). Even though individual low-quality patches, owing to their high extinction and low colonization rates, are only able to support transient occupancy (figure 4; electronic supplementary material, figures S4–S7), the presence of low-quality habitat facilitates more widespread susceptible persistence for a greater range of pathogens (figure 1). This phenomenon represents the other side of the role played by high-quality patches in that low-quality patches are relatively unstable (i.e. small local population sizes and high extinction rates) and thus do not support infectious populations for long. Since they are infrequently colonized (when high-quality habitat is preferentially selected, *ξ*_im_>0), the environmental reservoir left behind by these infectious populations probably decays before it has the opportunity to infect new susceptible colonists. As a result, low-quality patches effectively represent a dead-end for the pathogen, reducing the number of patches from which it can spread, and allowing susceptible hosts to more easily persist and avoid infection throughout the metapopulation. The opportunity for pathogen reservoir decay provided by the ephemeral occupancy of low-quality habitat echoes the ‘migratory escape’ hypothesis [20], which suggests that annual migration allows hosts to vacate contaminated sites and return once they are clean. Taken with this literature, our results highlight the importance of transient/temporary habitat in mitigating host exposure to environmentally transmitted pathogens.

### 4.3 Consequences for management

These results have general management implications that highlight the potential importance of low-quality habitat for the persistence of wildlife populations and the potential importance of high-quality habitat for the persistence of wildlife disease. It is critical to understand these implications in order to avoid falling into the management trap created by high-quality habitat and its interaction with pathogen spread. Previous theoretical work suggests that management should focus on maintaining patches where conditions are most favourable for the host (i.e. high-quality habitat), but Strasser *et al.* [21] show that stochastic disturbance (e.g. disease-induced mortality) can lead to cases where focusing on low-quality habitat is more effective in increasing population growth rate. Ovaskainen & Hanski [22] similarly demonstrate that correlated extinctions reduce the contributions of well-connected habitat to metapopulation persistence and increase the contributions of more isolated habitat. Our model adds to this literature by demonstrating the potential for environmentally persistent pathogens to provide the kind of disturbance under which low-quality habitat is beneficial and should thus be conserved.

In addition to helping to guide the prioritization of conservation efforts, our model may also help to inform the deployment of disease control measures, including the treatment of the environmental reservoir itself. In the case of African herbivores and their tick-borne pathogens, for example, the environmental reservoir created by ticks and livestock hosts could be controlled through the treatment of cattle with acaricides [23]. Our work suggests that such efforts would probably be most effective if focused on cattle that share high-quality habitat with the wildlife hosts of interest.

The framework used here provides general insights, but generating predictions for specific systems requires a careful consideration of the model’s simplifying assumptions. In particular, the SPOM, though well suited for capturing realistic spatial structure, does not account for within-patch dynamics. As a result, patches are modelled as either unoccupied or occupied by a host population with a fixed size and discrete disease status—either susceptible or infected. This occupancy framework assumes that within-patch dynamics (i.e. growth to carrying capacity and local pathogen spread) are rapid relative to among-patch dynamics (i.e. colonization and extinction). Moreover, it assumes that the pathogen spreads widely within a patch and affects host dynamics primarily by reducing population size. These are often reasonable assumptions, especially for relatively small populations in a highly fragmented landscape [12], but may not capture the nonlinearities and nuances of host–pathogen dynamics in a single population.

In addition, we assume that patch quality only influences population size and the resulting colonization and extinction processes, but quality could also influence pathogen dynamics within a patch through effects on host density or condition [24]. Patch quality may also influence the size or infectiousness of the environmental reservoir, either directly, by influencing the decay rate of the pathogen in the environment, or indirectly, through the effects on population size and overall shedding rate. More generally, the dynamics and establishment of environmental reservoirs could be influenced by a number of other factors, such as the length of infectious occupancy or the occupancy history of a patch. The potential importance of these additional mechanisms remains an open question for future work and needs to be considered before applying this framework to any given system of interest. Fortunately, the SPOM framework is fairly flexible and future work could extend the model presented here to include additional processes, e.g. by making transmission parameters also a function of patch quality.

Although detailed predictions for specific systems will probably require additional model development, the three scenarios identified here nonetheless provide a coarse framework that we can use to identify the potential risk of management traps in wildlife populations currently affected by an environmentally persistent pathogen. For instance, high-quality habitat seems unlikely to form management traps for bat populations affected by white nose syndrome owing to the widespread infection both within and between hibernaculum and the scarcity of host refuges [25,26]. Similarly, from these results, we would not expect the spread of chronic wasting disease to turn high-quality habitat into traps for mule deer metapopulations, owing again to widespread infection (albeit at low prevalence) across population units [27] and the relative importance of local-scale transmission within winter ranges [28]. However, we might expect to see high-quality traps in the plague-prairie dog system, where the infection is widespread and disease-induced mortality is high, but uninfected colonies are still able to persist [29]. Indeed, studies have suggested that large colonies are more likely to become infected [30], and thus high-quality habitat capable of supporting large populations may help facilitate plague persistence and spread. Thus, by considering the characteristics of a host–pathogen system and their effect on the roles played by high- and low-quality habitat, we can begin to diagnose where empirical systems fall relative to these three scenarios and the corresponding consequences for management.

## 5. Conclusion

This partitioning of pathogen dynamics into three regions based on whether high-quality habitat is a net benefit or detriment to the host metapopulation echoes the work of [31–33]. In particular, for a given pathogen, Hess [31] similarly identified three regions of disease dynamics which determined whether increased movement had a positive or negative effect on host occupancy. In cases where the host movement rate was either low enough that the pathogen was unable to invade or high enough to cause a widespread infection, Hess found that further increasing movement increased host occupancy, while at intermediate levels that resulted in moderate disease spread, increasing movement decreased host occupancy. These three scenarios, distinguished in [31] by the host movement rate, map to the three scenarios we outline here based on disease parameters (infectious survival and direct and environmental transmission). The conceptual similarity of these results suggests more generally that in metapopulations facing intermediate pathogen spread, factors that would normally increase host stability and connectivity in the absence of disease (e.g. increased movement, high-quality habitat) can actually decrease total host population size as a result of increased pathogen spread.

Given that the presence of this ecological trap depends on the relative magnitude of pathogen spread, we find that pathogens with long-lived environmental reservoirs only generate traps when infectious survival is low enough that the pathogen is not able to spread widely. Because environmentally long-lived pathogens are able to spread and persist more easily, increasing longevity can overwhelm the ability of low-quality habitat to slow spread and provide refuges for susceptible hosts. These dynamics reflect the findings of Gog *et al.* [32], who found that under strong background infection from an alternative host, increasing movement always increased total occupancy, despite facilitating pathogen spread. Park [33] expanded on this by adding environmental transmission to the mix and found that in cases where background and environmental transmission were strong relative to direct transmission, increasing movement was again a net benefit to the host metapopulation. These studies, coupled with the results presented here, suggest that when there is a persistent source of infection throughout the metapopulation (e.g. from long-lived environmental reservoirs), factors that facilitate metapopulation stability (e.g. increased movement, high-quality habitat) are generally a greater benefit than factors that inhibit pathogen spread (e.g. decreased movement, low-quality habitat).

## Supplementary Material

Supplementary figures Analogous versions of figures 1, 2, and 3 from the main text, but for a fully connected metapopulation (where every patch is equally accessible from every other patch), along with figures showing within patch occupancy for fixed disease parameters for both lattice and fully connected metapopulations.

## Data accessibility

Simulation source code and generated data are available on Github and archived at http://dx.doi.org/10.5281/zenodo.46194.

## References

[1] Miller MW, Hobbs NT, Tavener SJ 2006 Dynamics of prion disease transmission in mule deer. *Ecol. Appl.* 16, 2208–2214. (doi:10.1890/1051-0761(2006)016%5B2208:DOPDTI%5D2.0.CO;2) 17205898 10.1890/1051-0761(2006)016[2208:dopdti]2.0.co;2

[2] Dragon DC, Rennie RP 1995 The ecology of anthrax spores: tough but not invincible. *Can. Vet. J.* 36, 295–301. 7773917 PMC1686874

[3] Eisen RJ *et al.* 2008 Persistence of *Yersinia pestis* in soil under natural conditions. *Emerg. Infect. Dis.* 14, 941–943. (doi:10.3201/eid1406.080029) 18507908 10.3201/eid1406.080029 PMC2600287

[4] Reynolds HT, Ingersoll T, Barton HA 2015 Modeling the environmental growth of *Pseudogymnoascus destructans* and its impact on the white-nose syndrome epidemic. *J. Wildl. Dis.* 51, 318–331. (doi:10.7589/2014-06-157) 25588008 10.7589/2014-06-157

[5] Almberg ES, Cross PC, Johnson CJ, Heisey DM, Richards BJ 2011 Modeling routes of chronic wasting disease transmission: environmental prion persistence promotes deer population decline and extinction. *PLoS ONE* 6, e19896 (doi:10.1371/journal.pone.0019896) 21603638 10.1371/journal.pone.0019896 PMC3094393

[6] Sharp A, Pastor J 2011 Stable limit cycles and the paradox of enrichment in a model of chronic wasting disease. *Ecol. Appl.* 21, 1024–1030. (doi:10.1890/10-1449.1) 21774409 10.1890/10-1449.1

[7] Breban R, Drake JM, Stallknecht DE, Rohani P 2009 The role of environmental transmission in recurrent avian influenza epidemics. *PLoS Comput. Biol.* 5, e1000346 (doi:10.1371/journal.pcbi.1000346) 19360126 10.1371/journal.pcbi.1000346 PMC2660440

[8] George DB, Webb CT, Pepin KM, Savage LT, Antolin MF 2013 Persistence of black-tailed prairie-dog populations affected by plague in northern Colorado, USA. *Ecology* 94, 1572–1583. (doi:10.1890/12-0719.1) 23951717 10.1890/12-0719.1

[9] Moilanen A, Hanski I 1998 Metapopulation dynamics: effects of habitat quality and landscape structure. *Ecology* 79, 2503–2515. (doi:10.1890/0012-9658(1998)079[2503:MDEOHQ]2.0.CO;2)

[10] Robertson B, Hutto R 2006 A framework for understanding ecological traps and an evaluation of existing evidence. *Ecology* 87, 1075–1085. (doi:10.1890/0012-9658(2006)87[1075:AFFUET]2.0.CO;2) 16761584 10.1890/0012-9658(2006)87[1075:affuet]2.0.co;2

[11] Paull SH, Song S, McClure KM, Sackett LC, Kilpatrick AM, Johnson PTJ 2012 From superspreaders to disease hotspots: linking transmission across hosts and space. *Front. Ecol. Environ.* 10, 75–82. (doi:10.1890/110111) 23482675 10.1890/110111 PMC3589764

[12] Hanski I, Ovaskainen O 2003 Metapopulation theory for fragmented landscapes. *Theor. Popul. Biol.* 64, 119–127. (doi:10.1016/S0040-5809(03)00022-4) 12804876 10.1016/s0040-5809(03)00022-4

[13] Ovaskainen O, Hanski I 2004 Metapopulation dynamics in highly fragmented landscapes. In *Ecology, genetics, and evolution of metapopulations* (eds I Hanski, OE Gaggiotti), pp. 73–104. Amsterdam, The Netherlands: Elsevier Academic Press.

[14] R Core Team. 2014 *R: a language and environment for statistical computing*. Vienna, Austria: R Core Team. See http://www.R-project.org/.

[15] Gillespie D 1977 Exact stochastic simulation of coupled chemical reactions. *J. Phys. Chem.* 93555, 2340–2361. (doi:10.1021/j100540a008)

[16] Leach C, Webb C, Cross P 2016 Metapopulation disease spread simulation code. See http://dx.doi.org/10.5281/zenodo.46194. 10.1098/rsos.160051 PMC4821283 27069672

[17] Christley RM *et al.* 2005 Infection in social networks: using network analysis to identify high-risk individuals. *Am. J. Epidemiol.* 162, 1024–1031. (doi:10.1093/aje/kwi308) 16177140 10.1093/aje/kwi308

[18] Keeling MJ, Eames KTD 2005 Networks and epidemic models. *J. R. Soc. Interface* 2, 295–307. (doi:10.1098/rsif.2005.0051) 16849187 10.1098/rsif.2005.0051 PMC1578276

[19] Lloyd-Smith JO, Schreiber SJ, Kopp PE, Getz WM 2005 Superspreading and the effect of individual variation on disease emergence. *Nature* 438, 355–359. (doi:10.1038/nature04153) 16292310 10.1038/nature04153 PMC7094981

[20] Loehle C 1995 Social barriers to pathogen transmission in wild animal populations. *Ecology* 76, 326–335. (doi:10.2307/1941192)

[21] Strasser CA, Neubert MG, Caswell H, Hunter CM 2010 Contributions of high- and low-quality patches to a metapopulation with stochastic disturbance. *Theor. Ecol.* 5, 167–179. (doi:10.1007/s12080-010-0106-9)

[22] Ovaskainen O, Hanski I 2003 How much does an individual habitat fragment contribute to metapopulation dynamics and persistence? *Theor. Popul. Biol.* 64, 481–495. (doi:10.1016/S0040-5809(03)00102-3) 14630484 10.1016/s0040-5809(03)00102-3

[23] Keesing F, Allan BF, Young TP, Ostfeld RS 2013 Effects of wildlife and cattle on tick abundance in central Kenya. *Ecol. Appl.* 23, 1410–1418. (doi:10.1890/12-1607.1) 24147412 10.1890/12-1607.1

[24] Becker DJ, Streicker DG, Altizer S 2015 Linking anthropogenic resources to wildlife-pathogen dynamics: a review and meta-analysis. *Ecol. Lett.* 18, 483–495. (doi:10.1111/ele.12428) 25808224 10.1111/ele.12428 PMC4403965

[25] Langwig K *et al.* 2015 Host and pathogen ecology drive the seasonal dynamics of a fungal disease , white-nose syndrome. *Proc. R. Soc. B* 282, 20142335 (doi:10.1098/rspb.2014.2335) 10.1098/rspb.2014.2335 PMC4286034 25473016

[26] O’Regan SM, Magori K, Pulliam JT, Zokan MA, Kaul RB, Barton HD, Drake JM 2015 Multi-scale model of epidemic fade-out : will local extirpation events inhibit the spread of white-nose syndrome? *Ecol. Appl.* 25, 621–633. (doi:10.1890/14-0417.1) 26214909 10.1890/14-0417.1

[27] Conner MM, Miller MW 2004 Movement patterns and spatial epidemiology of a prion disease in mule deer population units. *Ecol. Appl.* 14, 1870–1881. (doi:10.1890/03-5309)

[28] Farnsworth ML, Hoeting JA, Hobbs NT, Miller MW 2006 Linking chronic wasting disease to mule deer movement scales: a hierarchical Bayesian approach. *Ecol. Appl.* 16, 1026–1036. (doi:10.1890/1051-0761(2006)016[1026:LCWDTM]2.0.CO;2) 16827000 10.1890/1051-0761(2006)016[1026:lcwdtm]2.0.co;2

[29] Stapp P, Antolin MF, Ball M 2004 Patterns of extinction in prairie dog metapopulations: plague outbreaks follow El Nino events. *Front. Ecol. Environ.* 2, 235–240. (doi:10.2307/3868263)

[30] Snäll T, O’Hara RB, Ray C, Collinge SK 2008 Climate-driven spatial dynamics of plague among prairie dog colonies. *Am. Nat.* 171, 238–248. (doi:10.1086/525051) 18197776 10.1086/525051

[31] Hess G 1996 Disease in metapopulation models: implications for conservation. *Ecology* 77, 1617–1632. (doi:10.2307/2265556)

[32] Gog J, Woodroffe R, Swinton J 2002 Disease in endangered metapopulations: the importance of alternative hosts. *Proc. R. Soc. Lond. B* 269, 671–676. (doi:10.1098/rspb.2001.1667) 10.1098/rspb.2001.1667 PMC1690941 11934357

[33] Park AW 2012 Infectious disease in animal metapopulations: the importance of environmental transmission. *Ecol. Evol.* 2, 1398–1407. (doi:10.1002/ece3.257) 22957148 10.1002/ece3.257 PMC3434925

