# Supplementary material for: When environmentally persistent pathogens transform good habitat into ecological traps
Source: R Soc Open Sci. 2016 Mar 23;3(3):160051. doi: 10.1098/rsos.160051 (PMC4821283; doi:10.1098/rsos.160051)
Supplement: Supplementary figures Analogous versions of figures 1, 2, and 3 from the main text, but for a fully connected metapopulation (where every patch is equally accessible from every other patch), along with figures showing within patch occupancy for fixed disease parameters for both lattice and fully con [file rsos160051supp1.pdf]

Supplementary figures

January 21, 2016

Supplemental figures 1, 2, and 3 are analogous to figures 1, 2, and 3 from the main text, but with all habitat patches equally connected (i.e. each habitat patch is equally accessible from all other habitat patches).

Supplemental figures 4 and 5, and 6 and 7 show within-patch occupancy for the lattice model and fully connected models, respectively, both with a fixed ν and δ.

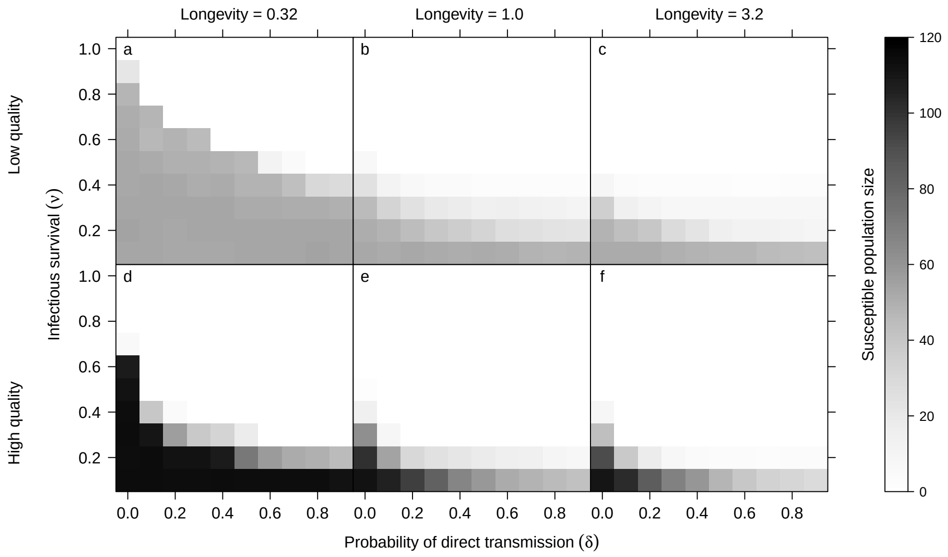

Figure 1: Median total susceptible population size as a function of infectious survival (ν), and the probability of direct transmission (δ). Panel columns show low (0.3, a, d), medium (1.0, b, e), and high (3.2, c, f) pathogen longevities, while rows show low quality (ranging from 0.23 to 1.24, a - c) and high quality (ranging from 0.76 to 1.77, d - f) patch quality distributions. Darker shading corresponds to larger population sizes, while white indicates that no susceptible host populations persist (i.e. all host populations become infected).

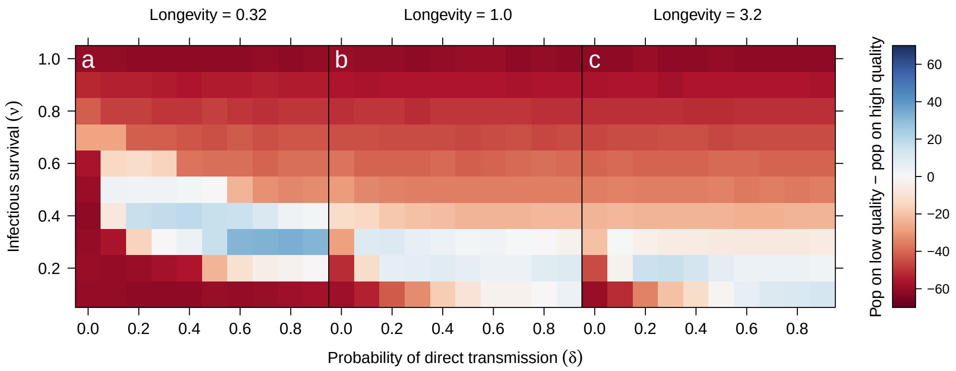

Figure 2: Differences between the median total population size of hosts on high quality patch distributions (quality between 0.76 and 1.77) and low quality patch distributions (quality between 0.23 to 1.24) as a function of infectious survival (ν) and the probability of direct transmission (δ). Reds indicate higher median host population sizes in high quality patch distributions, while blues indicate higher median host population sizes in low quality patch distributions. Panel columns show low (0.3, a), medium (1.0, b), and high (3.2, c) pathogen longevities.

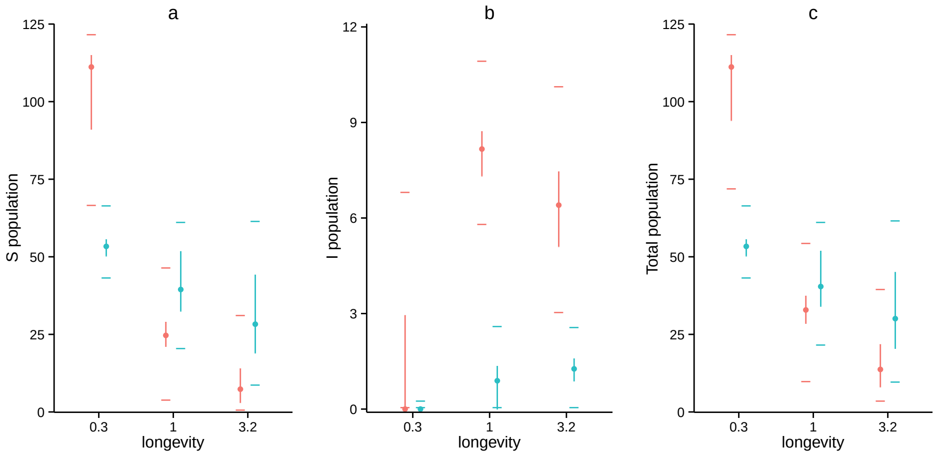

Figure 3: Boxplots showing the range, over 100 replicate simulations, of susceptible (a), infectious (b), and total (c) host population sizes for high quality (red) and low quality (blue) habitat distributions. Infectious survival (ν) and direct transmission rate (δ) are fixed at values of 0.2 and 0.3, respectively. Coloured points show medians, while vertical lines indicate the inner-quartile ranges, with horizontal lines indicating the minimum and maximum. At low longevities, this pathogen is unable to invade and high quality habitat supports larger populations of susceptible hosts. However, low quality habitat is better able to maintain these susceptible hosts as the pathogen's longevity increases, leading to larger total population sizes.

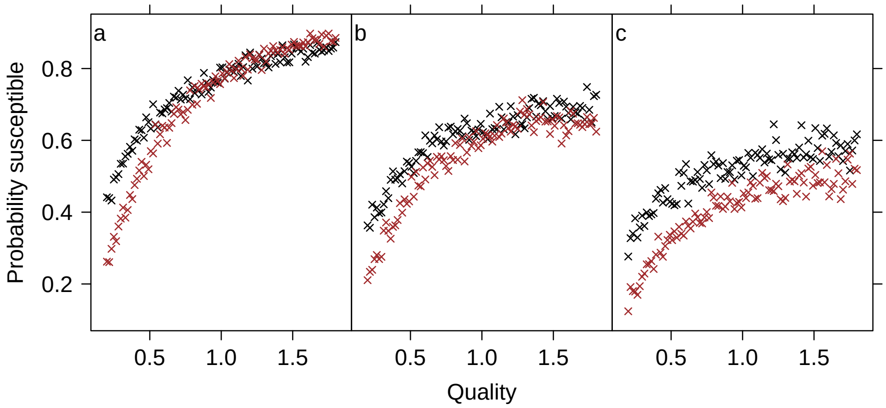

Figure 4: Mean probability of susceptible occupancy (measured as the proportion of time spent occupied by susceptible hosts over the last 500 time steps) as a function of patch quality. Red points indicate host preference for high quality habitat ($\xi_{im} = 0.5$), while black points indicate no preference ($\xi_{im} = 0$). Panel columns show low (0.3, a), medium (1.0, b), and high (3.2, c) pathogen longevities. Infectious survival and probability of direct infection both fixed at $\nu = 0.2$ and $\delta = 0.5$, with lattice landscape structure.

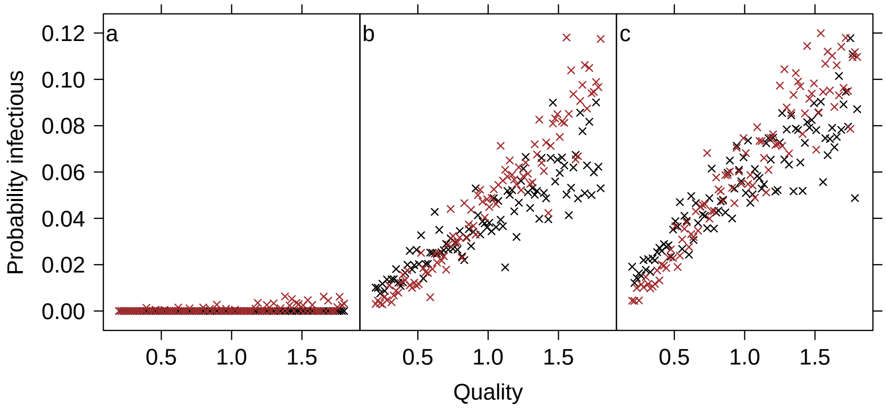

Figure 5: Mean probability of infectious occupancy (measured as the proportion of time spent occupied by infectious hosts over the last 500 time steps) as a function of patch quality. Red points indicate host preference for high quality habitat ($\xi_{im} = 0.5$), while black points indicate no preference ($\xi_{im} = 0$). Panel columns show low (0.3, a), medium (1.0, b), and high (3.2, c) pathogen longevities. Infectious survival and probability of direct infection both fixed at $\nu = 0.2$ and $\delta = 0.5$, with lattice landscape structure.

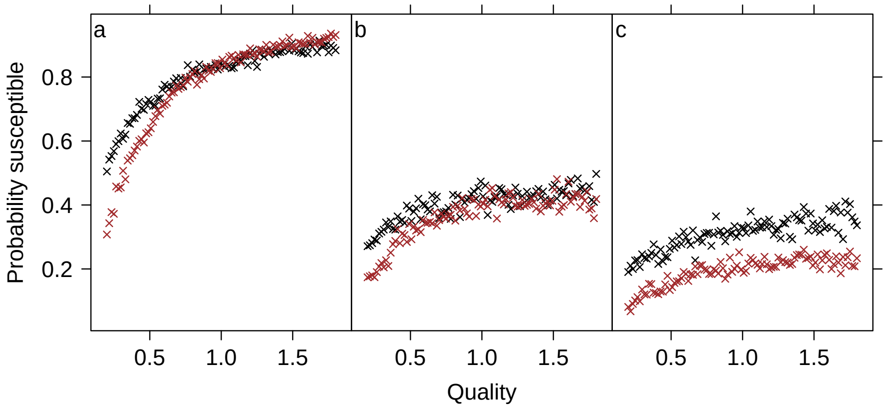

Figure 6: Mean probability of susceptible occupancy (measured as the proportion of time spent occupied by susceptible hosts over the last 500 time steps) as a function of patch quality. Red points indicate host preference for high quality habitat ($\xi_{im} = 0.5$), while black points indicate no preference ($\xi_{im} = 0$). Panel columns show low (0.3, a), medium (1.0, b), and high (3.2, c) pathogen longevities. Infectious survival and probability of direct infection both fixed at $\nu = 0.2$ and $\delta = 0.3$, with fully-connected landscape structure.

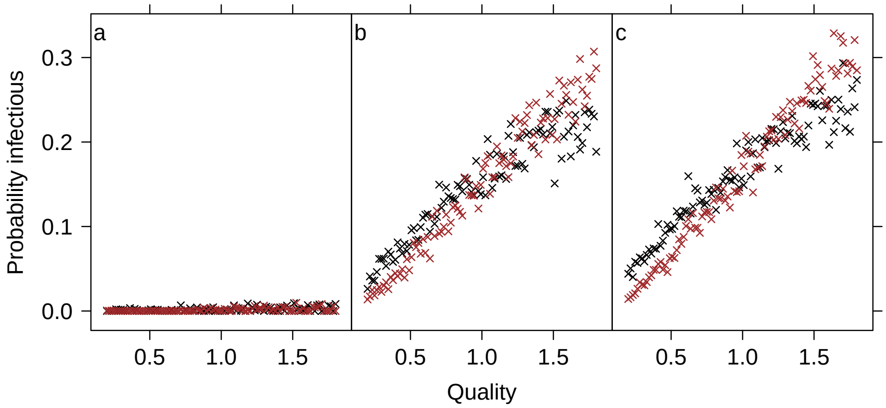

Figure 7: Mean probability of infectious occupancy (measured as the proportion of time spent occupied by infectious hosts over the last 500 time steps) as a function of patch quality. Red points indicate host preference for high quality habitat ($\xi_{im} = 0.5$), while black points indicate no preference ($\xi_{im} = 0$). Panel columns show low (0.3, a), medium (1.0, b), and high (3.2, c) pathogen longevities. Infectious survival and probability of direct infection both fixed at $\nu = 0.2$ and $\delta = 0.3$, with fully-connected landscape structure.
